# Pellitorine, a Potential Anti-Cancer Lead Compound against HL60 and MCT-7 Cell Lines and Microbial Transformation of Piperine from *Piper Nigrum*

**DOI:** 10.3390/molecules15042398

**Published:** 2010-04-02

**Authors:** Gwendoline Cheng Lian Ee, Chyi Meei Lim, Mawardi Rahmani, Khozirah Shaari, Choon Fah Joseph Bong

**Affiliations:** 1 Department of Chemistry, Faculty of Science, Universiti Putra Malaysia, 43400 Serdang, Selangor, Malaysia; 2 Department of Crop Science, Faculty of Agriculture & Food Sciences, Universiti Putra Malaysia Bintulu Campus, 97008, Bintulu, Sarawak, Malaysia

**Keywords:** *Piper nigrum*, pellitorine, alkaloids, cytotoxicity, microbial transformation, *aspergillus niger*

## Abstract

Pellitorine (**1**), which was isolated from the roots of *Piper nigrum*, showed strong cytotoxic activities against HL60 and MCT-7 cell lines. Microbial transformation of piperine (**2**) gave a new compound 5-[3,4-(methylenedioxy)phenyl]-pent-2-ene piperidine (**3**). Two other alkaloids were also found from *Piper nigrum.* They are (*E*)-1-[3’,4’-(methylenedioxy)cinnamoyl]piperidine (**4**) and 2,4-tetradecadienoic acid isobutyl amide (**5**). These compounds were isolated using chromatographic methods and their structures were elucidated using MS, IR and NMR techniques.

## 1. Introduction

**Figure 1:**
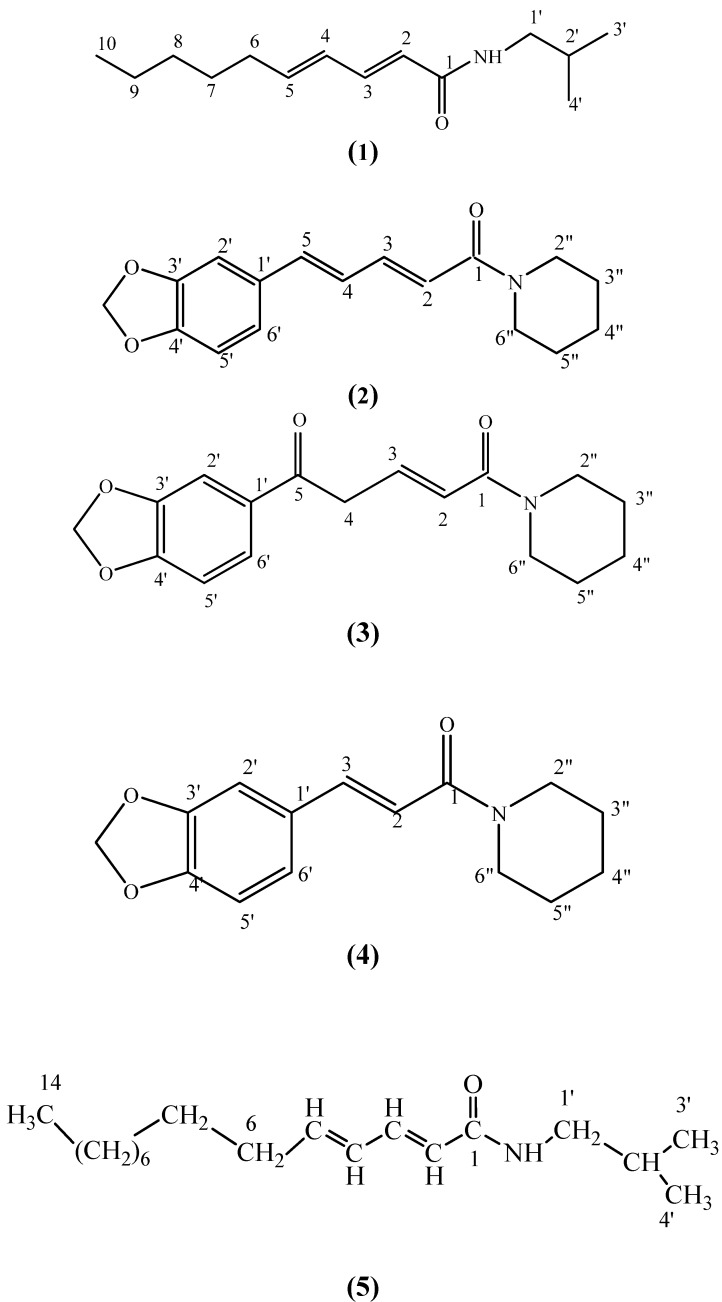
Compound structures.

The genus *Piper* is from the Piperaceae family which has over 700 species distributed in both hemispheres. The Piperaceae family is a source of many biologically active phytochemicals with great potential for medicinal and agricultural uses. Species in the genus *Piper* contain a wide array of secondary metabolite compounds, principally alkaloids and amides [1]. *Piper nigrum* is one of the more well-known species because of its high commercial, economic, and medicinal properties. It is known that *Piper nigrum* has biological activities such as CNS stimulant, analgesic, antipyretic and antifeedent activities [2]. This paper reports the isolation of the alkaloidal component pellitorine (**1**) and its cytotoxic activity against two cancer cell lines and the microbial transformation of piperine (**2**) into 5-[3,4-(methylenedioxy)phenyl]-pent-2-ene piperidine (**3**), which is a new compound (Figure 1). 

## 2. Results and Discussion

Pellitorine (**1**) was isolated as white crystals, with melting point 60–62 ºC (literature 69 ºC [3]). The spectral data are in agreement with published data [3]. Compound **3** was obtained as a yellow oil from the microbial transformation of piperine by *Aspergillus niger*. The EIMS displayed a molecular ion (M^+^) peak at m/z 301 and together with its ^1^H- and ^13^C-NMR data, indicated the molecular formula to be C_17_H_19_NO_4_. The molecular mass of **3 **also indicated an additional oxygen atom to be present in the molecule in comparison with piperine (2). The ^1^H-NMR spectrum of **3 **exhibited a methylene type signal at δ 3.89 (2H, m) which shifted from δ 6.74 (1H, m) that was assigned to a methine signal in the starting material. Meanwhile, the signal which appeared at δ 6.75 (1H, m) in the spectrum of piperine (**2**) was not seen in the ^1^H-NMR spectrum of this compound. Hence, it was deduced that this compound has only one aliphatic double bond as compared to piperine (**2**) which has two aliphatic double bonds. The ^13^C-NMR spectrum suggested the C-4 signal of piperine (**2**) at δ 124.87 was replaced by the signal at δ 45.96 in the spectrum of the product **3** and was confirmed by DEPT analysis. The carbon signal at δ 138.52 (C-5) shifted downfield to δ 169.12. This suggests that there is in existence another carbonyl group in this compound. In the HMBC spectrum, a *^2^J* correlation was observed between δ 45.96 (C-4) and δ 6.82 (H-3) and also between δ 45.96 (C-4) and δ 6.26 (H-2) via a *^3^J* correlation. The placement of the keto group at C-5 (δ169.12) was established from the HMBC experiment. The carbonyl signal at δ 169.12 showed correlations with the methylene at δ 3.89 through a *^2^J* coupling. Furthermore, comparison of the ^1^H- and ^13^C-NMR spectral data of **3 **with those of piperine permitted the assignment of **3 **as 5-[3,4-(methylenedioxy)phenyl]-pent-2-ene piperidine, which is a newly reported compound. The NMR data for **3** is tabulated in Table 1. 

**Table 1:** NMR data for **3**.

Position	^1^H NMR, δ ppm	^13^C NMR, δ ppm	HMBC
1		164.95	
2	6.26 (1H, d, *J*=13.7 Hz)	120.43	164.95 (C-1) (^2^J), 142.59(C-3) (^2^J), 46.96(C-4) (^3^J)
3	6.82 (1H, m)	142.59	164.95 (C-1) (^3^J), 120.43 (C-2) (^2^J), 46.96 (C-4) (^2^J)
4	3.89 (2H, m)	46.96	120.43 (C-2) (^3^J), 142.59(C-3) (^2^J), 169.12(C-5) (^2^J), 131.05 (C-1’) (^3^J)
5		169.12	
1’		131.05	
2’	6.69 (1H, s)	105.36	131.05 (C-1’) (^2^J), 122.12 (C-6’) (^3^J)
3’ & 4’	-	147.55	
5’	6.56 (1H, d, *J*=8.2 Hz)	108.10	131.05 (C-1’) (^3^J), 122.12 (C-6’) (^2^J)
6’	zhzhzzzz	122.12	108.10 (C-5’ )(^2^J)
OCH_2_O	6.61 (1H, d, *J*=2.7 Hz)	100.88	147.55 (C-3’) (^3^J), 147.55 (C-4’) (^3^J)
2”	5.86 (2H, s)	43.06	25.54 (C-2”) (^2^J), 25.54 (C-3”) (^3^J)
3” & 5”	3.40 (2H, m)	25.54	43.06 (C-1”) (^2^J), (^3^J)
4”	1.57 (4H, m)	26.56	25.54 (C-3” ) (^2^J), 25.54 (C-2”) (^3^J)
6”	1.57 (2H,m)	46.36	26.56 (C-4”) (^2^J)
	3.40 (2H, m)		

The structures of the other two alkaloids were determined by comparisons of spectral data with that of published data for (*E*)-1-[3’,4’-[methylenedioxycinnamoyl] piperidine (**4**) [4] and 2,4-tetradecadienoic acid isobutyl amide (**5**) [5]. The petroleum ether extract of *Piper nigrum* was tested for cytotoxic activities against HL60 (Human promyelocytic leukemia cells) cell line and was found to have a very low IC_50_ value of 9.8 µg/mL. Pellitorine on the other hand was extremely cytotoxic with an IC_50_ value of 13.0 µg/mL against the same cell line. It however, gave a much lower IC_50_ value of 1.8 µg/mL against the MCF-7 (breast cancer) cell line. Hence pellitorine could be a potential anti-cancer hit compound. On the other hand, the petroleum ether could also provide potential anti-cancer activity against the HL60 (Human promyelocytic leukemia cells) cells due to synergistic effects of the bioactives present in the extract. 

## 3. Experimental

### 3.1. Plant material

The roots of *Piper nigrum* were collected from Sri Aman, Sarawak, Malaysia. 

### 3.2. General

Infrared spectra were measured in NaCl pellet on a Perkin-Elmer FTIR Spectrum BX spectrometer. EIMS were recorded on a Shidmazu GCMS-QP5050A spectrometer. NMR spectra were obtained using Unity INOVA 500 MHz NMR/JEOL 400 MHz FTNMR spectrometers with tetramethylsilane (TMS) as internal standard and deuterated chloroform as solvent. Ultraviolet spectra were recorded in CHCl_3_ on a Shidmazu UV-160A, UV-Visible Recording Spectrophotometer.

### 3.3. Extraction and isolation

The dry powdered roots of *Piper nigrum* (4.8 kg) was extracted repeatedly for more than 48 hours with petroleum ether (2.5 L) followed by ethanol (2.5 L). All the extracts were combined and dried *in vacuo*. The ethanol extract was then added to a large quantity of 5% aqueous hydrochloric acid (1 L). The acidic solution was then filtered through kieselghur to remove non-alkaloidal substances. The filtrate was then basified with concentrated ammonia solution to pH 10. The liberated alkaloids were extracted exhaustively with chloroform. The chloroform extract was then washed with distilled water and dried over anhydrous sodium sulphate. The acid-base treated ethanol extract was obtained by removing the solvent under reduced pressure. The extract after evaporation yielded an oily extract (0.53 g). The extract was chromatographed over silica gel (120–230 mesh) column and fractions were collected in 100 mL aliquots. Fractions 10–26 gave pellitorine (**1**, 5 mg). Purification and isolation of the petroleum ether extract gave piperine (**2**, 950 mg), (*E*)-1-[3’,4’-(methylenedioxy)cinnamoyl]piperidine (**4**, 13 mg) and 2,4-tetradecadienoic acid isobutyl amide (**5**, 8 mg).

### 3.4. Biotransformation

#### 3.4.1. Screening procedures

Cultivation medium consisting of (g/L) 4.0 g glucose, 1.8 g peptone, 2.0 g KH_2_PO_4_, 0.4 g NaNO_3_, 0.1 g KCl, 0.1 g MgSO_4_·7H_2_O and 0.004 g FeSO_4_·7H_2_O was added into water. The fungus was transferred from the slants to 250 mL Erlenmayer flasks, each containing 200 mL of the medium. Pre- incubation was performed at 20 ºC for 48 h on a rotary shaker (150 rpm), until the proper growth of the *Aspergillus niger* FTCC 5003 was achieved. Then portions of 20 mg of a substrate (piperine), dissolved in 1.0 mL of acetone, was added to the grown culture. Control cultivation with no substrate was also performed. The growth of fungus was monitored each day for 15 days. Each time 100 µL of sample was taken out and quantitatively analyzed by HPLC. The solvent system used was 30% water: 70% methanol and with the flow rate at 0.5 mL/min. 

#### 3.4.2. Preparative biotransformation

Preparative scale biotransformation of piperine by *Aspergillus niger* FTCC 5003 were carried out in 30 × 250 mL conical flasks. Piperine (600 mg) was dissolved in acetone (30 mL) and the solutions were evenly divided among the flasks. The incubations were carried out for 14 days using the method mentioned above.

#### 3.4.3. Extraction and purification of biotransformation products

The cultures were filtered and the filtrates were extracted with the same amounts of chloroform thrice. The organic phase was collected and concentrated *in vacuo*. The residues were applied to a silica gel column and eluted with hexane-ethyl acetate with addition of methanol. Piperine was biotransformed by *Aspergillus niger* FTCC 5003 into 5-[3,4-(methylenedioxy)phenyl]-pent-2-ene piperidine (**3**), which was identified on the basis of spectroscopic data.

Pellitorine (**1**). White crystals with melting point 60–62 ºC (Literature 69 ºC, [3]). Spectral data are in agreement with literature values [3].

Piperine (**2**). Yellow crystals, melting point 126–128 ºC (Literature 128–129 ºC [6]). Spectral data are in agreement with published data [6].

*5-[3,4-(Methylenedioxy)phenyl]-pent-2-ene piperidine* (**3**). Yellow oil. UV λ_max _nm (Chloroform, log ε): 320 (4.00), 300 (0.25), 275 (0.75). IR ν_max_ (cm^-1­^, NaCl): 2929 (CH stretching), 1619 (C=O), 1493, 1444, 1359. MS (m/z, rel.int.): m/z 301 [M^+^] (3.92%), 271 (2), 251 (2), 221 (2), 204 (2), 189 (17), 177 (22), 154 (12), 143 (100), 131 (6), 112 (15), 109 (7), 86 (33), 70 (19), 69 (31), 55 (33). NMR data (see Table 1).

*(E)-1-[3’,4’-[Methylenedioxycinnamoyl] piperidine* (**4**). White crystals with melting point 74–76 ºC Spectral data are in agreement with published data [4].

### 3.5. Cytotoxicity assay

The cytotoxicity assays were carried out using HL60 and MCT-7 cell lines obtained from the National Cancer Institute (Bethesda, MD , USA. The cells were cultured and maintained in growth medium as described earlier by Mackeen *et al*., [7]. The stock solution was prepared at a concentration of 10 mg/mL in DMSO or absolute ethanol. A serial dilution of stock solution in ROM I-1640 medium provided seven sample dilutions at different concentrations. Cytotoxic assays were performed in 96-flat bottom microwell plate. Each well was filled with 100 µL of exponentially growing cell suspension in complete growth medium at a concentration of 5 × 10^5^ cell/mL. Controls were made containing only untreated cell population. The assay for each concentration of sample was performed in triplicate and the culture plate was incubated for 3 days at 37 ºC, 5% CO_2_ and 90% humidity. After three days, the fraction of surviving cells were determined relative to the untreated cell population by using the colorimeter MTT (3-(4,5-dimethylthioazzol-2yl)-2-5-diphenyltetrazolium bromide) method. The cytotoxic dose that killed 50% (CD_50_) was determined from absorbance (OD) versus concentration curve.

## 4. Conclusions

Pellitorine, a potential anti-cancer lead compound, was isolated from *Piper nigrum* and a new derivative of piperine was transformed by the microbe *Aspergillus niger*. Two other alkaloids were also isolated. The structures of these compounds were elucidated by using spectroscopic methods. 
